# Combining clinical chemistry with metabolomics for metabolic phenotyping at population levels

**DOI:** 10.1007/s11306-025-02331-2

**Published:** 2025-08-29

**Authors:** Yun Xu, Ian D. Wilson, Royston Goodacre

**Affiliations:** 1 Centre for Metabolomics Research, Department of Biochemistry, Cell and Systems Biology, Institute of Systems, Molecular and Integrative Biology, University of Liverpool, BioSciences Building, Crown St, Liverpool, L69 7ZB UK; 2 Division of Systems Medicine, Department of Metabolism, Digestion and Reproduction, Imperial College London, Hammersmith Campus, London, W12 0NN UK

**Keywords:** Clinical chemistry, Metabotyping, Multivariate statistical analysis, Biomarker discovery, HUSERMET study

## Abstract

**Introduction:**

Untargeted metabolic phenotyping (metabolomics/metabonomics), also known as metabotyping, has been shown to be able to discriminate reliably between different physiological or clinical conditions. However, we believe that standard panels of routinely collected clinical and clinical chemistry data also have the potential to provide assay panels that complement metabotyping.

**Objectives:**

To test the above hypothesis and evaluate the use of multivariate statistical analyses to provided panels of clinical/clinical chemistry data measurements that predict the age, sex and body mass index (BMI) of 977 normal subjects and compare these predictions with results acquired by metabotyping on the same healthy individuals.

**Methods:**

Metabotyping involved serum metabolomics using gas chromatography-mass spectrometry (GC-MS) and liquid chromatography-mass spectrometry (LC-MS) previously reported in our HUSERMET study (Dunn et al., 2015), while clinical chemistry data were obtained in clinic for 19 measurements assessing liver and kidney function, blood pressure, serum glucose, cations, as well as lipids. Multivariate analyses involved using support vector machines, random forest and partial least squares, to predict sex, age and BMI. These models used as inputs: (i) the clinical chemistry data alone; (ii) three metabolomics datasets; (iii) combinations of clinical chemistry with the metabolomics data. Model predictions were rigorously validated using 1,000 bootstrapping re-sampling coupled with permutation tests.

**Results:**

Multivariate statistical analyses on the clinical chemistry data obtained for these healthy participants could be used to predict: their sex, based on creatinine; their age, based on systolic blood pressure, total serum protein and serum glucose; as well as BMI using alanine transaminase, total cholesterol (Total-c) to high-density lipoprotein cholesterol (HDL-c) ratio and diastolic blood pressure. Combining clinical chemistry and metabolomics data sets enhanced the predictions of these characteristics. Moreover, this powerful combination allowed for quantitative predictions of age and BMI.

**Conclusion:**

Multivariate statistical analysis on clinical chemistry data from the HUSERMET study obtained similar predictions of age, sex or BMI, compared to metabotyping using GC-MS and LC-MS. These predictions from clinical chemistry data were between 71 and 85% accurate (depending on the MVA used) and compared favourably with metabolomics (71–91 depending on analytical method). Combining clinical chemistry and metabolomics data sets enhanced the predictions of these characteristics to 77–93% accuracy, suggesting that this augmentation of methods may be a useful approach in the search for clinical biomarkers.

**Supplementary Information:**

The online version contains supplementary material available at 10.1007/s11306-025-02331-2.

## Introduction

Untargeted metabolic phenotyping (metabonomics, metabolomics) is a well-established technology that has been shown to be an effective method for the analysis of biological systems for the detection of metabolites (or panels of metabolites) as potential biomarkers. Application areas are numerous and include human health and disease, nutrition, pharmacology, toxicology (and the exposome), plant sciences and agriculture, ecology and environmental sciences, as well as microbiology, biotechnology and engineering biology (e.g., synthetic biology and metabolic engineering). Central to metabolomics, in the traditional untargeted version, is the use of information-rich metabolite detection technologies such as nuclear magnetic resonance (NMR) spectroscopy and mass spectrometry (MS), with the latter either used alone via direct infusion methods (DI-MS) or linked to gas or liquid chromatography (e.g., GC-MS (Fiehn 2016) and LC-MS (Gika et al., 2019)). Such methods thereby generate profiles of the metabolites (including lipids) that are characteristic of the samples being analysed.

Arguably a major focus of metabolomics has been to generate profiles from biofluids (e.g., blood, urine, sweat, saliva, tears) that allow stratification of humans into various predefined classes. These human metabolic phenotypes may be used to differentiate diseased individuals from healthy controls, monitor progression of disease in response to treatment or, in non-clinical situations to describe human intrinsic factors such as sex, age, body mass index (BMI) as well as other clinically useful demographic data.

The “HUSERMET” (**HU**man **SER**um **MET**abolome) project, began in 2005, involved the collection of blood serum samples from nominally healthy human subjects (i.e., no known medical conditions at the time of sampling) who were resident in the Greater Manchester area of the UK (Dunn et al., 2015). It represented one of the first large-scale studies specifically undertaken to provide metabolic phenotypes (metabotypes), rather than re-using existing collections of samples (or as an adjunct to conventional clinical or epidemiological investigations). During the study *ca*. 4000 samples were collected from adults (aged between 19 and 81) who had no known disease at the time of sampling. From these samples some 1,200 were analysed, using both GC-MS and ultra high performance lipid chromatography (UHPLC-MS) (Dunn et al., 2015), for a wide range of wide range of metabolites. In addition, a large subset of these samples was also analysed using a battery of standard clinical chemistry assays. In the original publication, it was clearly demonstrated that it was possible to train multivariate models—namely support vector machines (SVM), random forest (RF) and partial least squares discriminant analysis (PLS-DA)—with the metabolomics data generated using these GC-MS and LC-MS analyses and to predict a number of selected demographic traits, such as sex, age and BMI with highly satisfactory accuracies between 87 and 92% (Dunn et al., 2015). It was also shown that these traits could be linked to specific metabolites; for example, citric acid was associated with ageing and was shown to increase with the age of the participant. However, the state of development of analytical methodology at the time was not sufficiently advanced to provide in depth metabolic profiles simply because of the difficulty of determining metabolite identity, which was especially acute for UHPLC-MS. Considering the significant advances in metabolic/lipidomic capabilities that have occurred since the end of the HUSERMET project we recently began to examine the feasibility of reanalysing these samples to see what how the level of information recovery for such a sample set has improved over the last decade, and this work is now in progress.

**Fig. 1:**
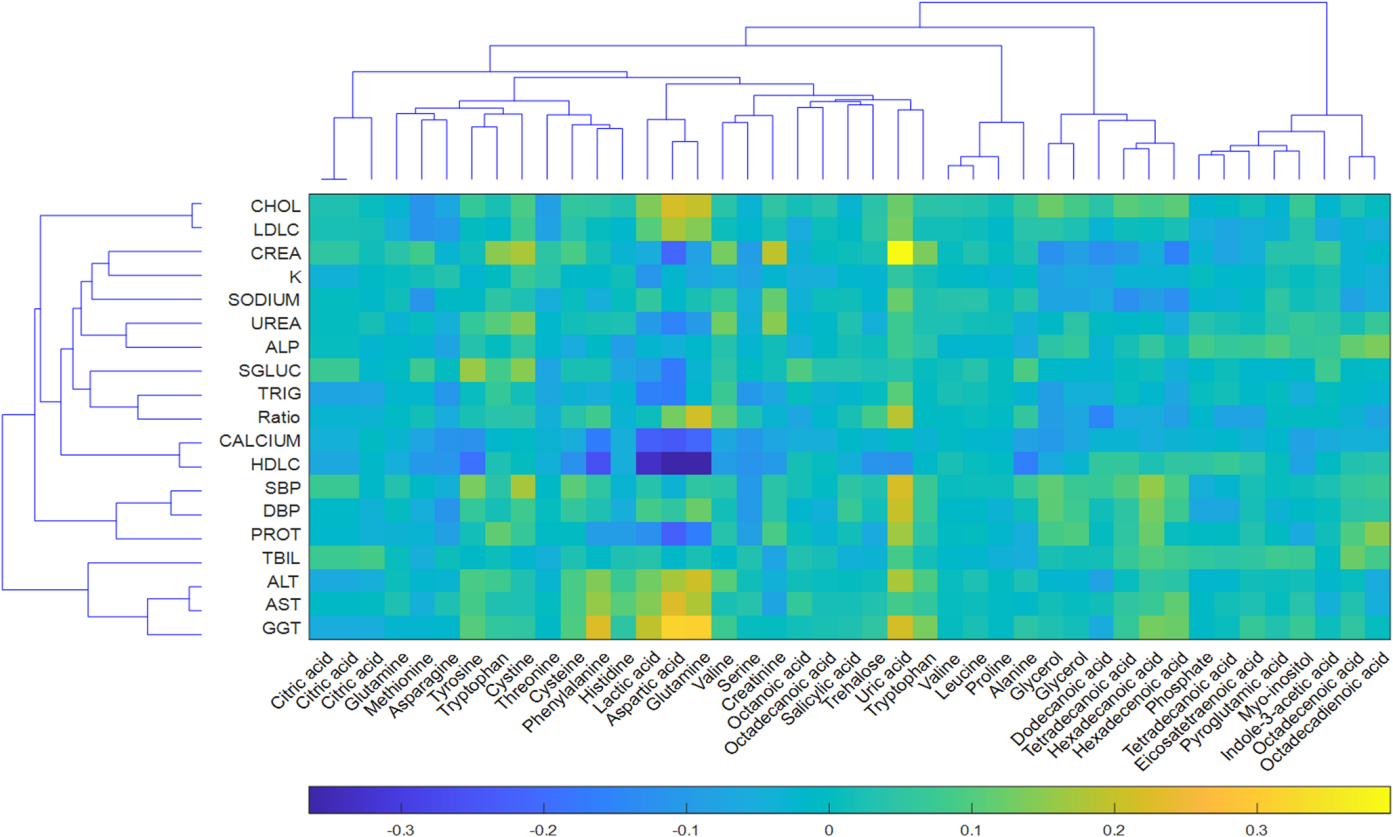
A heatmap, with dendrogram of Pearson’s correlation analysis for metabolites detected by GC–MS with MSI level of 1 (Sumner et al., 2007) and the 19 clinical chemistry measurements used in this study. The arrangement of rows (the clinical chemistry data) and columns (the GC-MS data) was produced by hierarchical clustering on both GC-MS metabolites and clinical chemistry data. The lower colour gradient bar represents coefficients from pairwise Pearson’s correlations (*R*) between the GC–MS and clinical chemistry data

However, concurrent with our above plans, as part of this re-examination of the HUSERMET samples, we have also conducted further work on the original data derived from the first investigation. One of the aspects we looked at was all of the demographic metadata obtained for each of the HUSERMET participants that had also been collected at the time sample collection. These included blood pressure, smoking status and a collection of clinical biochemistry assays such as alanine transaminase (ALT), aspartate transaminase (AST), glucose etc. One of the conclusions of this re-examination was that we had not sufficiently analysed these metadata, which had simply been used as supportive information at the time. Thus, while there are now a large number of publications using various types of “omics” data using a range of different statistical models to model and derive “biomarkers” none of these had ever been compared against models generated for the same subjects using multivariate models developed from standard clinical characteristics. This is perhaps surprising given that the later are generally routinely collected and much easier to obtain; moreover, huge amounts of historical data are already available. One possible reason for the lack of literature data is publication bias; i.e., such data may have indeed been examined but were found to be of little value and so were not submitted for publication. However, given the availability of both metabolic phenotyping and clinical chemistry data from HUSERMET, and the correlations that were visible in simple comparisons (as illustrated in Fig. 1; from a subset of samples (see below) obtained in the original work (Dunn et al., 2015), it seemed worth investigating the possible synergies between these different types of data.

To test the above conjectures the aim of the present study was to apply the same supervised learning models (*viz*., SVM, RF and PLS-DA) to test the hypothesis that the clinical chemical characteristic data would be able to predict one, or more, of three targets of interest (i.e., age, BMI and sex). These results were compared with those previously reported for the GC-MS and LC-MS metabolic phenotyping data (Dunn et al., 2015).

In addition, two types of chemometrics analyses were also conducted to further explore ways of analysing clinical chemistry data using multivariate approaches. One was data fusion where the clinical chemistry and GC-MS and LC-MS metabolomics data were combined using multiblock approaches to see if the two types of data can provide synergies when analysed together, thereby improving the overall results in the prediction of the age, BMI and sex of the subjects. A second type of analysis used *quantitative* predictions using partial least squares regression (PLS-R) analysis on the clinical chemistry data to predict the age and BMI of the subjects. These PLS-R predictions were also conducted on the metabolomics data for comparison with the predictions using clinical chemistry measures as the inputs, as well as combinations of both metabolomics and clinical measures.

## Materials and methods

### Metabolomics and clinical chemistry data

The original publications on the HUSERMET study, detailed in Dunn et al. (2015), contained metabolic profiles of serum obtained using GC-MS and LC-MS (in positive and negative electrospray ionisation modes) following our methods and protocols for large scale metabolomics (Begley et al., 2009; Zelena et al., 2009; Dunn et al., 2011). These original data comprised 1164 individuals with 25 clinical chemistry measurements made on the same subjects. The clinical chemistry parameters that were determined used methods that were standard at the time and may not reflect current practice.

The data set was not full in complement, and this was due to missing values in the clinical chemistry data. Therefore, *samples* with greater than a third of missing values were removed and *variables* with greater than a third of missing values were also removed. This left 977 individuals/samples with three sets of metabolomics data and 19 clinical chemistry measurements. The remaining missing values in the clinical chemistry data block (~ 6% of total values) were imputed using KNN imputation (Troyanskaya et al., 2001).

The clinical chemistry data contained: blood pressure (systolic and diastolic (SBP, DBP)), cations (Na^+^, K^+^, Ca^2+^), serum glucose, Total-c to HDL-c ratio (RATIO), liver function (ALP, ALT, AST, gamma-glutamyl transferase (GGT), bilirubin and total protein), kidney related measurements (creatinine, urea), as well as lipid-related measurements (cholesterol, triglycerides, high-density lipoprotein cholesterol and low-density lipoproteins cholesterol (HDLC, LDLC). The ranges of between 2.5 and 97.5% percentiles of these 19 measurements are present in Table 1, along with their definitions.

**Table 1:** The range of clinical chemistry measurements

Name	Median (2.5%– 97.5% percentile)	Standard deviation	Reference range
SBP (mm Hg)	125 (115-136)	16.17	<120
DBP (mm Hg)	76 (60-98)	10.21	<80
PROT (g L^−1^)	70 (68-73)	6.65	60-80
CREA (µmol L^−1^)	75.1 (51.0-106.7)	15.79	59-104 for men; 45-84 for women
SGLUC (mmol L^−1^)	4.7 (3.4-8.1)	1.19	3.9-5.5
SODIUM (mmol L^−1^)	141 (140-142)	2.11	135-145
K (mmol L^−1^)	4.3 (3.7-5.1)	0.35	3.5-5.3
CALCIUM (mmol L^−1^)	2.33 (2.17-2.50)	0.40	2.12-2.55
CHOL (mmol L^−1^)	5.1 (3.3-7.1)	1.01	<5.0
TRIG (mmol L^−1^)	1.2 (0.4-3.5)	1.04	<1.7
HDLC (mmol L^−1^)	1.25 (0.8-2.1)	0.74	>1 for men; >1.2 for women
LDLC (mmol L^−1^)	3.2 (1.5-5.1)	0.92	<2.6
RATIO	4.0 (2.2-7.3)	1.24	<6
UREA (mmol L^−1^)	5.2 (3.0-10.7)	1.89	2.5-7.8
TBIL (µmol L^−1^)	10.0 (5.0-25.0)	5.09	1.7-20.5
ALP (I.U. L^−1^)	68 (37-148)	25.63	30-130
ALT (I.U. L^−1^)	21 (10-68)	15.97	7-56
AST (I.U. L^−1^)	21 (14-42)	9.56	0-40
GGT (I.U. L^−1^)	20 (9-106)	24.00	5-40

*SBP* systolic blood pressure, *DBP* diastolic BP, *PROT* total serum protein, *CREA* serum creatinine, *SGLUC* serum glucose, *K* potassium, *CHOL* cholesterol, *TRIG* triglycerides, *HDLC* high-density lipoprotein cholesterol, *LDLC* low-density lipoprotein cholesterol, *RATIO* total cholesterol to HDL cholesterol ratio, *TBIL* total bilirubin, *ALP*, alkaline phosphatase, *ALT* alanine aminotransferase, *AST* aspartate aminotransferase, *GGT* gamma-glutamyl transferase

The GC-MS data set produced by Dunn et al. (2015) contained 126 detected metabolite *mz*/t_R_ features: 43 had MSI level 1 identifications, 19 were MSI level 2 and 64 were not identified (MSI level 4). The LC-MS + ve and -ve ESI data had 2178 and 2280 detected metabolite features respectively. For the LC-MS + ve ESI data, 659 features were categorized as being either MSI level 2 identification and the remaining 1519 were not identified and assigned MSI level 4; for the LC-MS -ve ESI data, 386 features were MSI level 2 with the remaining 1894 unidentified features assigned to MSI level 4.

### Statistical analysis

#### Univariate statistical testing

Wilcoxon rank sum test was applied to the clinical chemistry data to identify significant clinical chemistry features that were significantly different between male and female, two classes of age and BMI (see below for definitions). Benjamin-Hochberg procedure (Benjamin et al., 1995) was also applied to obtain false discovery rate of these features.

#### Comparison of clinical chemistry and metabolomics data

In order to compare the capability of both clinical chemistry and metabolomic data to predict the sex, age and BMI of subjects, we have re-analysed the three metabolomic data sets, GC-MS, UHPLC + ve and -ve electrospray ionisation (ESI) data, using the same subset of 977 subjects as reported in (Dunn et al., 2015) and described above. Three classification models, PLS-DA, SVM (with a linear kernel) and Random Forests (RFs) were used to predict the sex, age and BMI of these subjects. The same models and validation procedures were employed on the clinical chemistry data to predict the same three characteristics. For the metabolomic data sets, the same data-preprocessing procedure as described in (Dunn et al., 2011, 2015) were applied. For the clinical chemistry data set, following KNN imputed data were auto-scaled so that each variable had a mean of 0 and a standard deviation of 1 before subjected to classification modelling.

The age and BMI modelling were conducted in the same way as reported in the previous publication: for age modelling, it was defined as a two-class classification problem in which subjects were divided into two groups: those who were under 50 and those who were older than 65 while the data for subjects aged between 50 and 65 were ignored; in BMI modelling, the two classes were subjects with a BMI of over 30 and those with BMI no more than 25, while subjects with a BMI between 25 and 30 were ignored. It is worth noting that the definitions of classes for age and BMI were inherited from the previous study (Dunn et al., 2015) which is to ensure a compatible comparison. The definition itself is not necessarily optimal. For example, a recent study had suggested that the break point of aging may be at 44 and 60 (Shen et al., 2024), rather than 50 and 65 as used here.

All the models were validated using 1,000 bootstrap re-samplings coupled with permutation tests as described in (Xu & Goodacre, 2025). For each bootstrapping re-sampling, *n* (*n* was the number of subjects) subjects were sampled with replacement (i.e., the same subject could be sampled multiple times) and they were used as the training set whilst those who were not selected were used as the test set. Due to the random nature of bootstrapping re-sampling, the size of training and test set varied with an expectation that an average of 63.2% of the samples would be used for training and 36.8% would be used for testing (Gromski et al., 2015; Efron & Tibshirani, 1994). A classification model (the observed model) was trained on the training set and the model’s hyper-parameters (e.g., the number of PLS components in PLS-DA) were optimised using a 7-fold cross-validation procedure performed on the training set. The trained the model was then applied to the test to get classification performance statistics such as correct classification rate (CCR) and to generate a confusion matrix. In addition, within each bootstrapping re-sampling another model was also trained on the training set with their labels randomly permuted (NULL model). The NULL model was also applied to the test set and predictions of the NULL model were compared with the real models for the test sets only—in this process histograms are produced of the real *versus* null to see how strong the models were (Xu & Goodacre, 2025). Following this, an empirical *p*-value was derived by calculating the percentage of the cases when the performance of the NULL model was better than the corresponding observed model.

It is worth noting that for BMI and age binary classification (i.e., BMI < 25 *vs*. BMI > 30 and age < 50 *vs*. age > 65), the class distribution was heavily skewed. For BMI classification to discriminate subjects with BMI < 25 and those with BMI > 30, there were 421 subjects (71%) having BMI < 25 and 172 subjects (29%) have BMI > 30; for age classification to discriminate age < 50 vs. age > 65, there were 524 subjects (78%) younger than 50 and 148 subjects (22%) older than 65. Without intervention, the results would be skewed towards the major class (Fernandez et al., 2018). Therefore, during the training stage the two classes of age and BMI were balanced by randomly under-sampling (RUS) (Seiffert et al., 2009) the major classes so that there were equal numbers of samples for both. In addition, for these two types of modelling, balanced accuracies, defined as the average of true positive and true negative of each class (Fernandez et al., 2018), were calculated to measure the performance of the model instead of CCRs as CCR does not reflect the true performance in predicting the unknowns when the class distribution is skewed.

In the same way as studied in our previous publication (Dunn et al., 2015), the effect of sample size was also investigated by calculating the learning curve of each classification model. Instead of using bootstrapping re-sampling to split training and test sets in which the ratio of training and test set may vary, we randomly select 10–90% samples with a 10% interval as the training set and the remaining 90–10% samples were used as the test set. The same performance statistics were calculated on each combination of training and test set. This procedure repeated 100 times, and the performance statistics were averaged and plotted against the percentage of the samples used for training. This generated a learning curve showing how the model would be affected by increasing the numbers of samples for training.

#### Data fusion

The possibility of data fusion of the clinical chemistry and metabolomic data was also explored. Therefore, three *ad hoc* multiblock data matrices were prepared. In each multiblock data matrix, the clinical chemistry data matrix was concatenated with the GC-MS, +ve or -ve ESI UHPLC data matrices on row direction with the same subject was placed on same rows. Block scaling was then applied to each block (i.e., clinical chemistry matrix and one of the three metabolomic matrices) separately. In this type of analysis, the block was firstly mean centred and divided by the standard deviation of each variable, then the whole block was divided by the square root of the number of variables. This ensured that each block would have had the same variance, and that the model would not have been dominated by the block with highest number of variables. These three multiblock matrices were analysed using the same models and validation procedure as described above.

### Regression analysis of clinical chemistry data

**Fig. 2:**
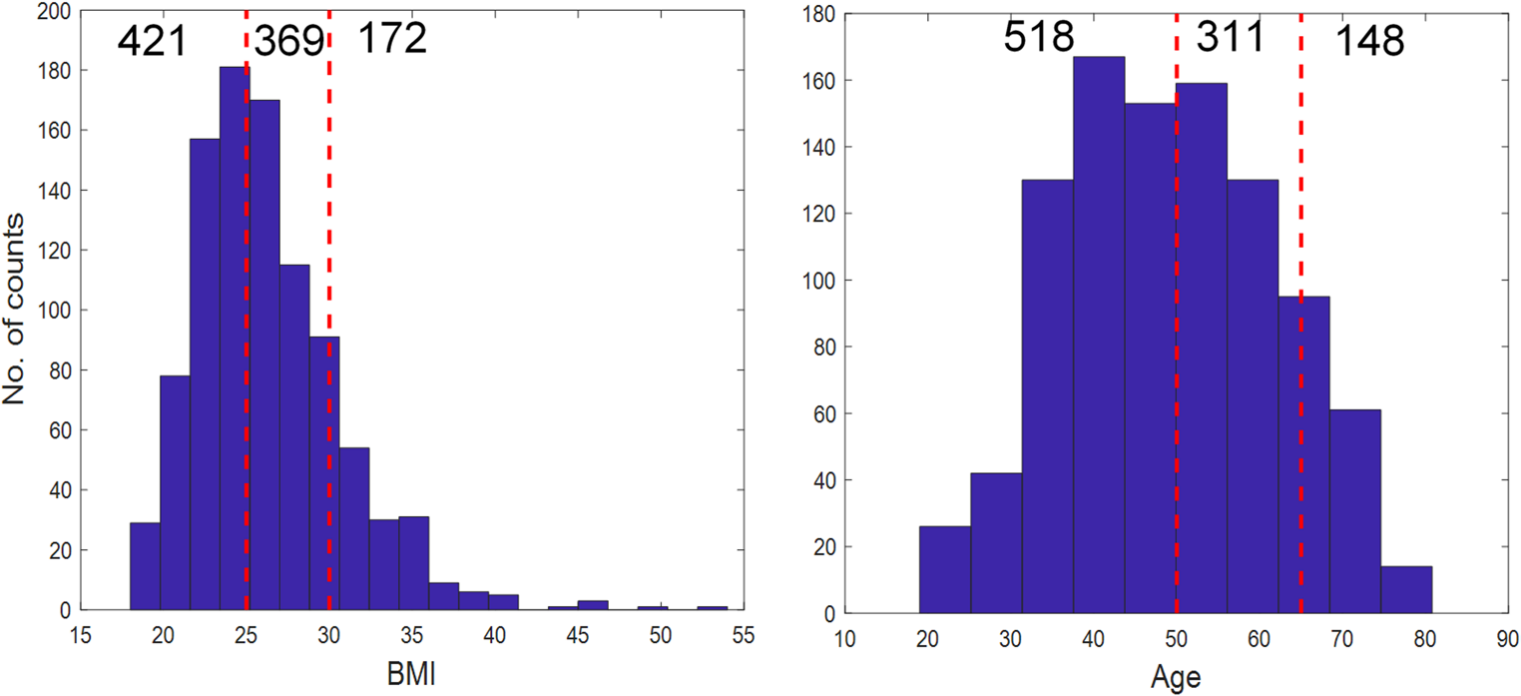
The distribution of BMI and age of the 977 subjects. The samples in the BMI range 25 to 30 and age range 50 to 65 which were not included in the original analysis of those data used to predict BMI (369) and age (311) are highlighted by red dotted lines

As described above and as preformed in the previous HUSERMET study (Dunn et al., 2015), age and BMI were arbitrarily classified as two groups: age < 50 *vs*. age > 65 and BMI < 25 *vs*. BMI > 30. This type of categorical prediction only made use of approximately 60–70% of the data as subjects with age between 50 and 65 or BMI between 25 and 30 were excluded from these data only for the reason of convenience of analysis. The distributions of age and BMI of the 977 subjects in the present study are shown in Fig. 2 to give a view of the number of samples that had not been used for age and BMI modelling in the previous study. Thus, to utilise the data available fully, and to access full potential of the clinical chemistry data for predicting age and BMI, further quantitative regression analysis was performed on the full datasets without leaving out those subjects aged between 50 and 65 or a BMI between 25 and 30.

Since both BMI and age can be considered as continuous variables partial least squares regression (PLS-R) was hereby employed in which the age and BMI of the subjects were used as the response variable. These models were also validated using 1,000 bootstrapping resampling with permutation tests, as described above except that figures of merit for regression were used to assess the performance of the model, namely root mean squares error of prediction (RMSEP) and validated coefficient of determination (*Q*^2^), which are defined as follows:

$$\:RMSEP=\:\sqrt{\frac{\sum\:_{i=1}^{n}{\left({y}_{i}-\widehat{{y}_{i}}\right)}^{2}}{n}}$$

$$\:{Q}^{2}=1-\frac{\sum\:_{i=1}^{n}{\left({y}_{i}-\widehat{{y}_{i}}\right)}^{2}}{\sum\:_{i=1}^{n}{\left({y}_{i}-\stackrel{-}{y}\right)}^{2}}$$

where $\:{y}_{i}$ is the known age or BMI of subject *i*; $\:\widehat{{y}_{i}}$ was the predicted age or BMI of subject *i*; $\:\stackrel{-}{y}$ was the averaged known age or BMI and *n* was the number of samples in the test set. RMSEP represents the expected absolute deviation from true value in predictions while *Q*^2^ represents the percentage of variance in the response variable (i.e., age or BMI) explained by the model. A perfect model would have a *Q*^2^ of 1 while a poor model can have *Q*^2^ significantly lower than 1, and can even be negative (i.e., the error of modelling is greater the total variance of the response variable). Both figures of merit were calculated using the predictions of the blind test set.

## Results and discussion

### Univariate tests on clinical chemistry data

The results of Wilcoxon rank sum tests are presented in Table S1 in ESI. Fifteen out of 19 clinical chemistry features obtained significant (< 0.05) FDR adjusted *p*-values between male and female. For both age differences (age < 50 *vs*. age > 65) and BMI differences (BMI < 25 *vs*. BMI > 30), 11 features obtained significant (< 0.05) FDR adjusted *p*-values.

### Comparison between clinical chemistry and metabolomic data

Having established in previous work that mass spectrometry-based metabolic profiling of human blood serum can be used to predict sex, age and BMI with high accuracies (Dunn et al., 2015), we aimed to test our hypothesis that similar predictions can be derived from the clinical chemistry data that were obtained from the same sample/person. Therefore, a series of models were generated using supervised learning algorithms including PLS-DA, SVM and RFs on just the 19 measurements from the clinical chemistry data from the 977 individuals from HUSERMET. Next three models were generated on the GC-MS data alone, LC-MS in the positive ion mode alone, LC-MS in the negative ion mode alone again from the same 977 individuals, thereby allowing direct comparisons. After which, these three different metabolic profiling data sets were combined with the clinical chemistry data as described in the Materials and Methods section above.

Below we briefly highlight the main findings in terms of the balanced accuracies from these models. Note that we only present data from the test set predictions, and that all these models have been validated using bootstrap resampling (*n* = 1000 models) along with 1000 corresponding NULL models from permuting the *Y*-variable.

**Fig. 3:**
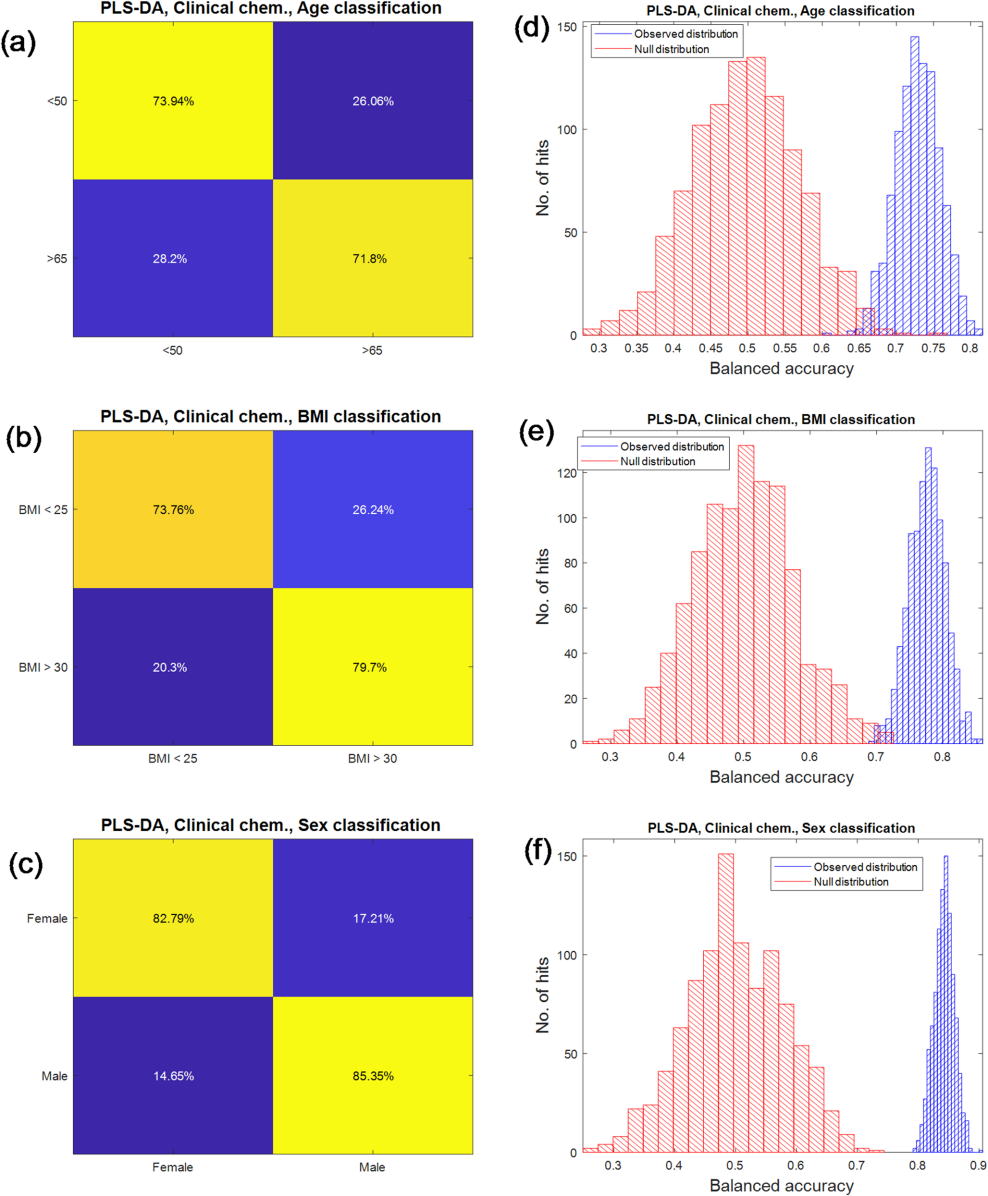
Confusion matrices (**a**–**c**) and distributions of the balanced accuracies of the observed models *vs*. NULL models (**d**–**f**) using PLS-DA model performed on just the clinical chemistry data for age, BMI and sex classification. The confusion matrices **a**–**c** show the expected positive and negative results in the columns and actual predicted positive and negative results in the rows. Thus the top left box is true positive (TP), top right false positive (FP), bottom left false negative (FN) and the bottom right box shows true negatives (TN). The values are the averages from the 1000 test sets and the boxes are coloured from yellow to blue, with yellow showing the largest percentage in blue the smallest percentage in that box. The histograms **d**–**f** are the corresponding balanced accuracy distributions for the test set predictions from the 1000 bootstraps for the real models in blue, and the NULL distribution from permutation testing in red

**Fig. 4:**
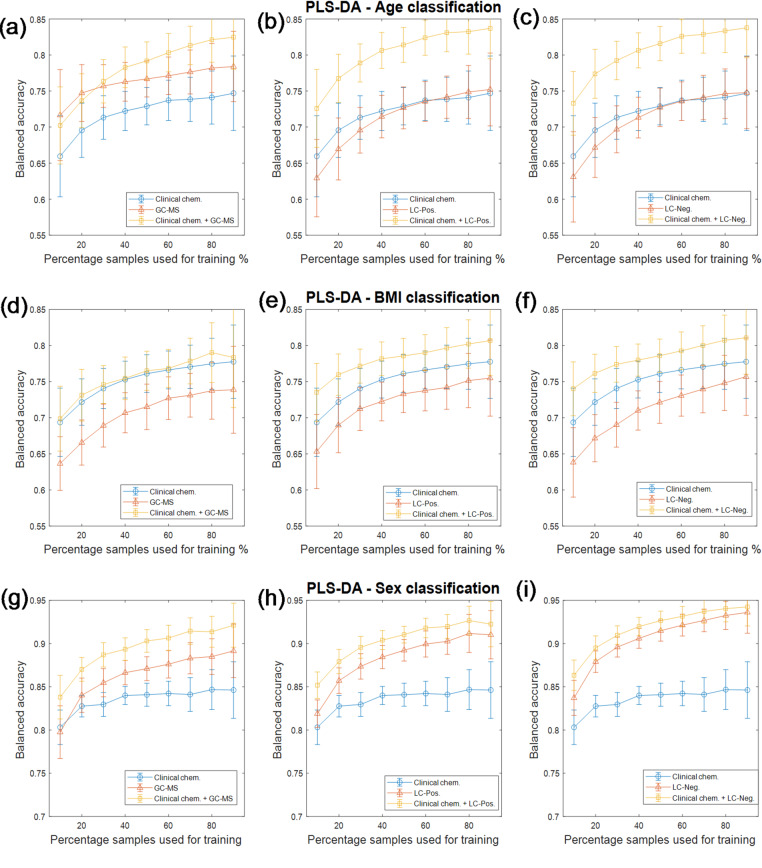
Learning curves of PLS-DA models: **a**–**c** are the learning curves for age classifications using GC-MS, LC-Positive mode and LC-Negative mode data respectively. The learning curves of these three types of data sets fused with clinical chemistry data are presented in the corresponding sub-figures respectively. Finally, the learning curve of the model performed on clinical chemistry data is presented in each of the sub-figure as well; **d**–**f** are the same type of learning curves for BMI classification and **g**–**i** are for sex classification

The averaged balanced accuracies of sex, age and BMI of the test sets generated by bootstrapping re-sampling are listed in Table 2(a). The confusion matrices, distributions of the performance of observed *vs*. NULL models of PLS-DA models performed on clinical chemistry data are presented in Fig. 3 while those of other models/data combinations are presented in Figures S1 to S11 in the supporting information (SI). The results of all three types of classifications using clinical chemistry data were comparable with only +/- 3% differences for most models. It is also interesting to see that there is not a single combination of a type of data and model that always outperform others in predicting ages, BMI and sex. For sex prediction, the best combination was SVM using -ve ESI UHPLC-MS data with 91.4% averaged balanced accuracy while the worst combination was RF using GC-MS data with only averaged 81.8% accuracy. The accuracy of the results of clinical chemistry data were between these two with 83.6–84.2% averaged balanced accuracy. For BMI prediction, the best combination was also SVM using -ve ESI UHPLC-MS data with 79.8% averaged balanced accuracy while the worst combination was SVM using GC-MS data with 71.1% averaged balanced accuracy. Clinical chemistry data performed well with PLS-DA with 76.7% averaged balanced accuracy which outperformed all three types of metabolomic data sets. It also outperformed GC-MS with SVM and random forest with 77.1% and 76.6% averaged balanced accuracy respectively, only slightly worse (< 2%) than the two UHPLC-MS data sets. For age predictions, the best results were achieved by SVM on the + ve ESI UHPLC-MS data with 81.2% averaged balanced accuracy. Models using clinical chemistry data were the worst for all three types of models. An interesting observation is that different models behaved differently when applied to different types of data. It seems that the results of PLS-DA performed on different data sets are more similar to each other, the clinical chemistry data had less than 2% averaged balanced accuracy compared to the three metabolomics data sets while for SVM and RF, approximately 9% gap had been observed between the best and worst performing models performed on four different types of data. However, all three classification models had much closer performance when applied to data sets fused with clinical chemistry data. In addition, the confusion matrices showed that the predictions of the RF approach were seemly still significantly skewed towards the major class, indicating that random under sampling had been less successful for this type of model while it appeared to be most successful when applied to PLS-DA models. The learning curves of PLS-DA performed on clinical chemistry data are presented in Fig. 4 and the learning curve of SVM and RF models are presented in Figure S22 and S23 respectively in SI. This analysis showed a more detailed picture of the models with different sizes of data used for training and testing. Overall, the performance of the models of the clinical chemistry data sets were comparable with the three metabolomic data sets on all percentages of samples used for training. However, it appeared that, compared to the metabolomics data, the clinical chemistry data benefited less from increasing the size of data used for training. It was often found that the performance of the models using clinical chemistry data performed as well as or better than the ones using metabolomics data. However, the learning curves for the clinical chemistry data plateaued quickly after approximately 30–40% of samples were used for training while the models made using metabolomics data continued to show improvements in performance with more samples available for training. Thus, towards the end of curve the models with metabolomics data often outperformed the ones constructed with similar amounts of clinical chemistry training data. This result is most likely due to the fact that the number of variables in the clinical chemistry data were much smaller than in the corresponding GC-MS and LC-MS data and thus models generated using it can generalize well, and reach optimal performance, with fewer samples. In addition, although their is a general trend that the model performance increased when more samples were used for training, when the percentage of training set was too high (e.g. 70–90%), the standard deviations were also increased significantly, suggesting high variation in performance estimations when different combinations of training and test set were used for modelling and validation. This implies that, in order to get a robust estimation of the performance of the models, a good balance between the size of training and that of test set is essential. This is consistent with our previous report based on simulation data (Xu & Goodacre, 2018).

**Table 2:** Averaged balanced prediction accuracies of two-class classifiers

(a): Averaged balanced accuracies of models trained on single data sets							
		Clinical Chemistry	GC-MS		LC-Pos.		LC-Neg.
Sex^1^							
PLS-DA		84.1%^***^	86.2%^***^		83.6%^***^		82.5%^***^
SVM		84.5%^***^	87.7%^***^		88.9%^***^		91.4%^***^
Random Forest		84.2%^***^	81.8%^***^		82.7%^***^		83.9%^***^
BMI^2^							
PLS-DA		76.7%^**^	73.7%^**^		75.1%^***^		74.1%^**^
SVM		77.7%^***^	71.1%^***^		77.9%^***^		79.8%^**^
Random Forest		76.6%^***^	73.1%^***^		78.5%^***^		77.9%^***^
Age^3^							
PLS-DA		72.2%^***^	75.6%^**^		74.4%^***^		73.4%^**^
SVM		72.3%^***^	74.5%^***^		81.2%^**^		80.6%^***^
Random Forest		71.3%^***^	76.7%^***^		80.7% ^***^		80.2%^***^

(b) Averaged balanced accuracies of models trained on metabolomic data sets combined with clinical chemistry data							
	Clinical Chemistry + GC-MS			Clinical Chemistry + LC-Pos.		Clinical Chemistry + LC-Neg.	
Sex							
PLS-DA	91.2%^***^			91.9%^***^		92.8%^***^	
SVM	90.6%^***^			90.6%^***^		92.1%^***^	
Random Forest	84.6%^***^			84.6%^***^		87.4%^***^	
BMI							
PLS-DA	78.2%^***^			79.5%^***^		79.5%^***^	
SVM	77.5%^***^			79.4%^***^		80.3%^***^	
Random Forest	77.0%^****^			80.1%^****^		79.4%^***^	
Age							
PLS-DA	81.4%^***^			83.2%^***^		81.4%^***^	
SVM	78.4%^***^			82.2%^***^		81.5%^***^	
Random Forest	80.5%^***^			82.1%^***^		82.0%^***^	

^1^Defined as male *versus* female

^2^Defined as < 25 BMI *versus* >30 BMI

^3^Defined as < 50 years *versus* >65 years

*: *p* < 0.05; **: *p* < 0.01; ***: *p* < 0.001

### PLS-R on full clinical chemistry data for age and BMI prediction

Having established that the clinical chemistry data did indeed have utility for predicting age, sex and BMI in this cohort, and that by combining these clinical chemistry data with MS-based metabolomics improved the predictions for the above classification models, we then tested whether age and BMI could be predicted in a *quantitative* manner—since it is reported as a continuum and any classification used is arbitrary. This had the added benefit that rather than discard some data (i.e., 311 individuals based on age and 369 people based on BMI) all 977 individuals could be used within the modelling (Fig. 2).

The results of PLS-R models for age and BMI predictions are listed in Table 3. Also, figures of known *vs*. predicted and distributions of observed *vs*. NULL distribution are shown in Figure S23 and S25 in SI. The results show that although all the models were statistically significant compared to NULL distributions, the performances of the model were moderate to poor with significant errors in predictions. This is not unexpected as there are significant variations between individuals and would certainly result in large variations in predictions for each single target value (e.g., age or BMI). However, such modelling has proved that there was a statistically significant correlation between the clinical chemistry, metabolomics data and the age and BMI of the subjects. Interestingly, for this type of modelling, the results derived from the clinical chemistry data were significantly better than those of metabolomics data. For BMI prediction, the median of *Q*^2^ (a figure of merit for linearity in terms of validated coefficient of determination) of clinical chemistry data was 0.2943 and the median error from RMSEP calculations was 3.7691. These results are significantly better than those of GC-MS (*Q*^2^ = 0.1388, RMSEP = 4.1746), +ve ESI UHPLC-MS (*Q*^2^ = 0.2057, RMSEP = 4.0112) and -ve ESI UHPLC-MS (*Q*^2^ = 0.2029, RMSEP = 4.0115). For age prediction, the median of *Q*^2^ and RMSEP of clinical chemistry data were 0.3423 and 10.2169 respectively which again was better than those for GC-MS (*Q*^2^ = 0.1460, RMSEP = 11.6652), +ve ESI UHPLC-MS (*Q*^2^ = 0.2638, RMSEP = 10.8171) and -ve ESI UHPLC-MS data (*Q*^2^ = 0.279, RMSEP = 10.7161). These findings have been tabulated in Table 1.

**Table 3:** PLS-R regression for age and BMI prediction

(a) Individual data sets					
	Clinical Chemistry	GC-MS		LC-Pos.	LC-Neg.
Age					
Median *Q*^2^	0.3423	0.1460		0.2638	0.2790
Median RMSEP	10.2169	11.6652		10.8171	10.7161
BMI					
Median *Q*^2^	0.2943	0.1388		0.2057	0.2029
Median RMSEP	3.7691	4.1746		4.0112	4.0115

(b) Metabolomics data combined with clinical chemistry data					
	Clinical Chemistry + GC-MS		Clinical Chemistry + LC-Pos.		Clinical Chemistry + LC-Neg.
Age					
Median *Q*^2^	0.4147		0.4976		0.5105
Median RMSEP	9.6336		8.9268		8.8144
BMI					
Median *Q*^2^	0.2656		0.3167		0.3617
Median RMSEP	3.8551		3.7030		3.5934

**Fig. 5:**
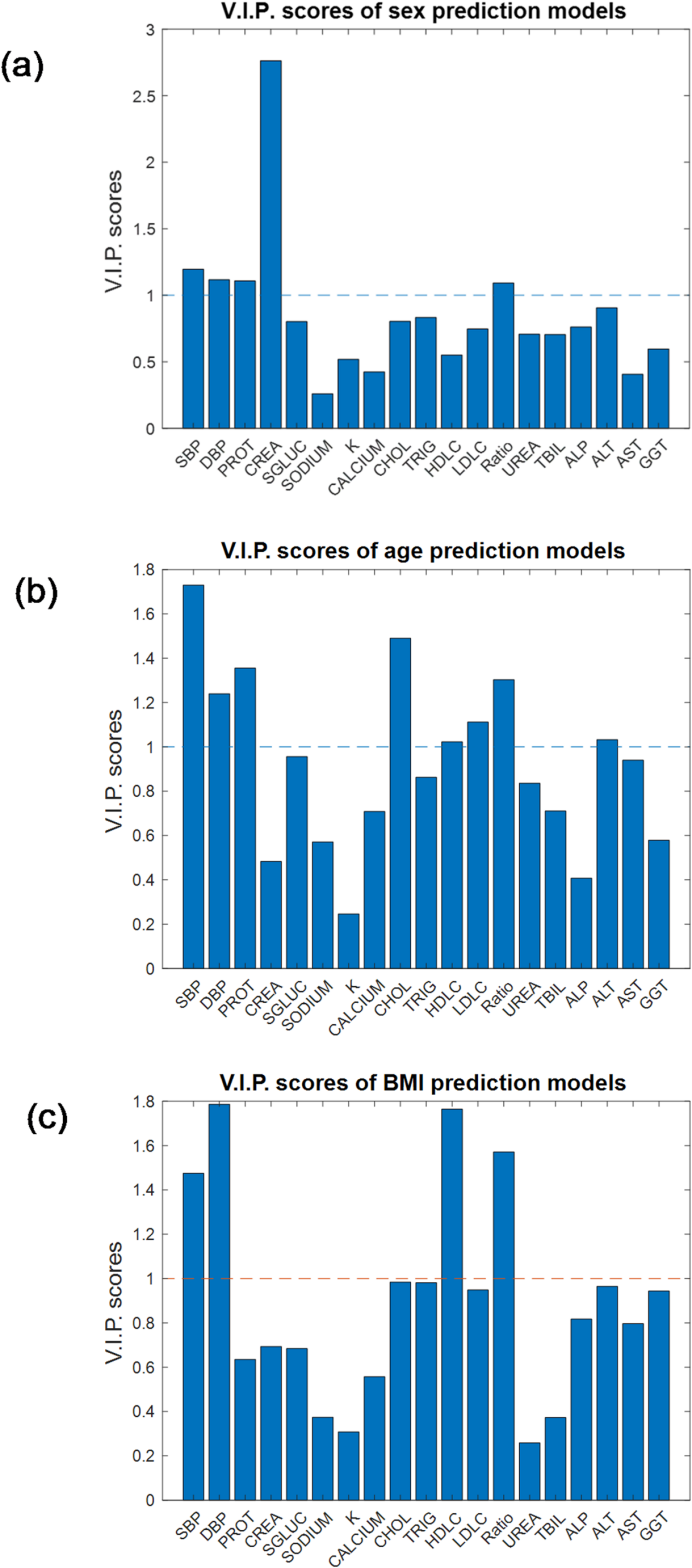
V.I.P. scores plots of **a** PLS-DA model for sex classification, **b** PLS-R model for age prediction and **c** PLS-R model for BMI prediction

**Fig. 6:**
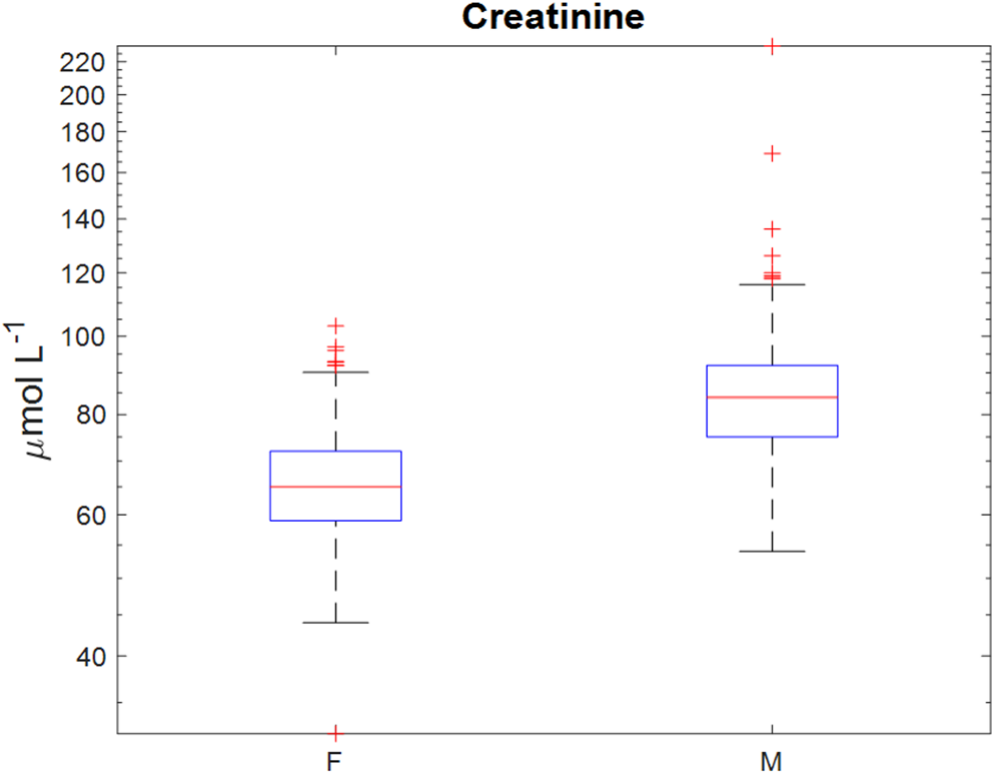
Box-whisker plot of serum creatinine concentrations, grouped by the sex of the subjects

A useful feature of multivariate modelling is that the models can provide useful statistics to identify which variables contributed most to the modelling and these may be the ones most important for discriminating between designated classes (for classification) or have the strongest correlation with the response variable (for regression). Therefore, Variable Importance in Projection (V.I.P.) scores from PLS were used to help identifying most important variables in clinical chemistry data set for age, BMI and sex predictions. To identify important features for the model using the V.I.P. values, the commonly used “greater than one rule” criterion was employed for significant feature identification, i.e. the features with the V.I.P. scores > 1 are considered as important (Chong et al., 2005). The V.I.P. scores plots of the PLS-DA model for sex classification and the PLS-R models for age and BMI predictions are shown in Fig. 5. The most predominant clinical chemistry variable for sex classification was creatinine (CREA), suggesting that there was a significant difference in serum creatinine concentrations between males and females. The FDR adjusted *p*-value of creatinine for male *vs*. female comparison using Wilcoxon rank sum test was also highly significant which is 1.04 × 10^−89^. The box-whisker plot (Fig. 6) has shown that the serum concentrations of creatinine determined using the standard clinical chemistry assay were indeed significantly lower in female subjects compared to males. It was less obvious for V.I.P. scores plots of PLS-R for age and BMI predictions. However, using a threshold of one, blood pressure (SBP and DBP), total Protein (PROT), cholesterol (CHOL), HDL-c (HDLC), LDL-c (LDLC), total-c to HDL-c ratio (Ratio) and ALT were identified as important for age prediction whilst SBP, DBP, HDLC and Ratio were important for BMI prediction.

### Data fusion

Finally, we investigated the potential of combing both the HUSERMET clinical chemistry and metabolic phenotyping data (*viz*., GC-MS, LC-MS + ve, LC-MS -ve) into single models via data fusion. The results of these classification models trained on multiblock matrices are listed in Table 2(b) and the corresponding confusion matrices, distributions of the performance of the observed and NULL models are presented in Figures S12 to S20 in SI. The results of PLS-R regression models trained on multiblock matrices are listed in Table 2(b), the known *vs*. predicted and observed *vs*. NULL distribution plots are presented in Figures S24 and S26 in SI. These results showed that, for both classification and regression, all models trained on multiblock matrices outperformed the corresponding models trained on individual metabolomic or clinical chemistry data. The gain in performance varied between combinations of data and models. For classification models, the most significant improvement was found in PLS-DA using -ve ESI UHPLC-MS data for sex prediction, with a 10.3% increase in balanced accuracy, while the least gain was found in RF, also using -ve ESI UHPLC-MS data, for BMI predictions, which gave only a 0.5% increase in balanced accuracy. Although such an increase seems minor, it was still the best performance achieved for BMI predictions. A general trend is that the ones with most gains from data fusion were usually those data/model combinations that had most underperformed compared to other combinations for the same type of classification. As a result, the gap between performance between data/model combinations for the same type of classification were much narrower than those obtained using single data sets. This suggests that data fusion can help to achieve the best result that the data can offer. Similarly, PLS-R models also benefit from data fusion with significant improvements observed in *Q*^2^ and RMSEP. For GC-MS data set, when it was “fused” with clinical chemistry data the *Q*^2^ increased from 0.1388 to 0.2656 for BMI predictions and from 0.1460 to 0.4147 for age predictions; for + ve ESI UHPLC-MS, the *Q*^2^ increased from 0.2057 to 0.3167 for BMI predictions and from 0.2638 to 0.4976 when it was “fused” with clinical chemistry data; finally the data fusion between -ve ESI UHPLC-MS data and clinical chemistry data increased the *Q*^2^ from 0.2029 to 0.3617 for BMI predictions and from 0.2790 to 0.5105 for age predictions. Overall, models for age predictions obtained greater gains from data fusion than those for BMI predictions and the results were much better. This is mainly because that the distribution of BMI was more left-skewed and there were very few subjects had high BMIs (> 30). The known vs. predicted plots for BMI models showed that the predictions of samples with high BMIs were all significantly deviated from known values and thus resulted in low *Q*^2^ and high RMSEP. Such plots for age predictions are more balanced around the diagonal *y = x* line where the cases of predicted = known should be.

## Conclusions

The results of the multivariate statistical analysis of the clinical chemistry data obtained from the participants in the HUSERMET study for the prediction of sex, age and BMI were found to be comparable with those previously obtained (using same modelling methods) for GC-MS and UHPLC-MS-derived metabolomic data (Dunn et al., 2015), and the subset of 977 analysed in the present study. Interestingly, this multivariate analysis of the clinical chemistry data highlighted relatively specific “panels” of clinically relevant analytes for each of these physiological states, with relatively little overlap between them. In the case of the prediction of sex, creatinine provided one such “biomarker” based on differences in serum concentrations being lower in females than males. Now, this difference was already well known to clinical chemists who had a need to generate reference values against which to compare patients for the diagnosis of kidney disease. In a statistical study of data obtained for serum creatinine (Pottel et al., 2008) noted that it increased for both girls and boys during childhood but between 20 and 70 years of age remained reasonably stable for both sexes. Above this age serum creatinine again began to increase slightly, probably as a result of declining renal function. However, it is likely that there are also variations in serum creatinine (Scr) concentrations for individuals of different ethnicities not just based on their sex (Pottel et al., 2012). The age of an individual could be predicted from the clinical chemistry data based on systolic blood pressure (SBP), total protein (PROT) and serum glucose (SGLUC), all with smaller contributions from other analytes. For BMI the model the most significant “biomarkers” were alanine transaminase (ALT), and diastolic blood pressure (DBP). The acquisition of such data are, as a result of the widespread availability of clinical analysers and routine measurements of blood pressure, easier to perform compared to the more specialized instrumentation used in metabolomics and would enable individuals to be easily placed physiologically within their population (especially if these data were concatenated to give a more holistic picture of the individual). With this information an investigator could highlight individuals that fell outside the normal ranges for their age or sex could be identified and perhaps investigated further, using more biochemically informative omic techniques, such as metabolomics, where metabolites have also been associated with e.g., age, sex (Pottel et al., 2012) and obesity (e.g., Oberbach et al., 2017; Park et al., 2015) and HUSERMET (Dunn et al., 2015). Last but not least, the most important features identified by the multivariate models (i.e. V.I.P. scores) also obtained most significant *p*-values in Wilcoxon rank sum tests (Table S1), showing good consistencies between the two types of significant feature identification methods. However, there are also a some interesting contradictions between the V.I.P. scores and Wilcoxon-Rank sum tests which is HDLC and Ratio (total-c to HDL-c ratio). Both features obtained insignificant FDR (> 0.1). However, both had obtained significant V.I.P. scores (> 1), indicating they made significant contributions in discriminating subjects with age < 50 and those with age > 65. Since these two measurements are inherently correlated, it highlighted a limitation of univariate test which is that it cannot take interactions between features into account and thus may miss important features (Sun et al., 1996). On the other hand, there were also numerous cases where the features obtained highly significant features, but they did not get significant V.I.P. scores, suggesting that feature selection only by multivariate modelling also have high chance missing significant features. Therefore, it is a good practice to conduct both multivariate model based feature selection and univariate statistical testing (Xu et al. 2025).

It is clear that the information content of HUSERMET clinical chemistry data set, analysed here using multivariate statistical techniques, provides information that is greater than the sum of its parts. This opens up the intriguing possibility of initially “mining” the huge amounts of clinical chemistry data available using multivariate modelling to improve disease diagnosis and provide more personalised healthcare (see for example the illustration provided by Cohen et al., 2021). However, this does not mean that the metabolic phenotyping data are irrelevant to this process as it was also noteworthy that the fusion of both types of data resulted in stronger predictions of sex, age and BMI characteristics and would probably do the same for improving disease diagnosis and personalized medicine, hopefully with an added layer of biochemical understanding. Indeed, we found that by combining clinical chemistry data with metabolic profiling from LC-MS (in either ionisation mode (Figure S24) we were able to predict age quantitatively. This opens up exciting avenues in terms of measuring biological/metabolic ageing, rather than relying on chronological age.

The original HUSERMET study showed, via multivariate supervised modelling, that there were correlations between the metabolomic data and the results of standard clinical chemistry. Multivariate statistical analysis of the metabolomics data from HUSERMET was found to be able to predict age, BMI and sex with high accuracy. The current multivariate statistical analysis of these clinical chemistry data from the HUSERMET study was able to achieve similar levels of predictivity for these characteristics. Moreover, data fusion between clinical chemistry and metabolomics data provided universal improvements in prediction accuracies. This provides a demonstration that both clinical chemistry and metabolomic data can provide complementary information and there may be value in using this approach the routinely collected clinical chemistry data even in the absence of “omic” studies. In conclusion, the combination of both metabolomics and clinical chemistry data gave the best results and there may be potential the same approach, for example, when seeking biomarkers in clinical conditions.

## Supplementary Information

Below is the link to the electronic supplementary material.

Supplementary Material 1

## Data Availability

Data are provided within the manuscript or supplementary information files.

## References

[CR1] Begley, P., Francis-McIntyre, S., Dunn, W. B., Broadhurst, D. I., Halsall, A., Tseng, A., Knowles, J., Consortium, H. U. S. E. R. M. E. T., Goodacre, R., & Kell, D. B. (2009). Development and performance of a gas chromatography – time-of-flight mass spectrometry analysis for large-scale nontargeted metabolomic studies of human serum. *Analytical Chemistry,**81*, 7038–7046. 10.1021/ac9011599 19606840 10.1021/ac9011599

[CR2] Benjamini, Y., & Hochberg, Y. (1995). Controlling the false discovery rate: A practical and powerful approach to multiple testing. *Journal of the Royal Statistical Society Series B*, *57*, 289–300. 10.1111/j.2517-6161.1995.tb02031.x

[CR3] Chong, I. G., & Jun, C. H. (2005). Performance of some variable selection methods when multicollinearity is present. *Chemometrics and Intelligent Laboratory Systems*, *78*, 103–112. 10.1016/j.chemolab.2004.12.011

[CR4] Cohen, N. M., Schwartzman, O., Jaschek, R., Lifshitz, A., Hoichman, M., Balicer, R., Shlush, L. I., Barbash, G., & Tanay, A. (2021). Personalized lab test models to quantify disease potentials in healthy individuals. *Nature Medecine*, *27*, 1582–1591. 10.1038/s41591-021-01468-6 10.1038/s41591-021-01468-6 34426707

[CR5] Dunn, W. B., Broadhurst, D., Begley, P., Zelena, E., Francis-McIntyre, S., Anderson, N., Brown, M., Knowles, J. D., Halsall, A., Haselden, J. N., Nicholls, A. W., Wilson, I. D., The Husermet consortium, Kell, D. B., & Goodacre, R. (2011). Procedures for large-scale metabolic profiling of serum and plasma using gas chromatography and liquid chromatography coupled to mass spectrometry. *Nature Protocols,**6*, 1060–1083. 10.1038/nprot.2011.335 21720319 10.1038/nprot.2011.335

[CR6] Dunn, W. B., Lin, W., Broadhurst, D., Begley, P., Brown, M., Zelena, E., Vaughan, A. A., Halsall, A., Harding, N., Knowles, J. D., Francis-McIntyre, S., Tseng, A., Ellis, D. I., O’Hagan, S., Aarons, G., Benjamin, B., Chew-Graham, S., Moseley, C., Potter, P., Winder, C. L., Potts, C., Thornton, P., McWhirter, C., Zubair, M., Pan, M., Burns, A., Cruickshank, J. K., Jayson, G. C., Purandare, N., Wu, F. C. W., Finn, J. D., Haselden, J. N., Nicholls, A. W., Wilson, I. D., Goodacre, R., & Kell, D. B. (2015). Molecular phenotyping of a UK population: defining the human serum metabolome *Metabolomics* 11, 9–26. 10.1007/s11306-014-0707-1 10.1007/s11306-014-0707-1 PMC4289517 25598764

[CR7] Efron, B., & Tibshirani, R. (1994). *An introduction to the bootstrap*. Chapman & Hall/CRC. 10.1201/9780429246593

[CR8] Fernández, A., García., S., Galar, M., Prati., R. C., Krawczyk, B., &amp; Herrera, F. (2018). *Learning from imbalanced data sets*. Springer Nature.

[CR9] Fiehn, O. (2016). Metabolomics by gas Chromatography-Mass spectrometry: The combination of targeted and untargeted profiling. *Current Protocols in Molecular Biology*, *114*. 30.4.1–30.4.32. 10.1002/0471142727.mb3004s114 10.1002/0471142727.mb3004s114 PMC4829120 27038389

[CR10] Gika, H., Virgiliou, C., Theodoridis, G., Plumb, R. S., & Wilson, I. D. (2019). Untargeted LC/MS-based metabolic phenotyping (metabonomics/metabolomics): The state of the art. *Journal of Chromatography B,**1117*, 136–147. 10.1016/j.jchromb.2019.04.009 10.1016/j.jchromb.2019.04.009 31009899

[CR11] Gromski, P. S., Muhamadali, H., Ellis, D. I., Xu, Y., Correa, E., Turner, M. L., & Goodacre, R. (2015). A tutorial review: Metabolomics and partial least squares-discriminant analysis—a marriage of convenience or a shotgun wedding. *Analytica Chimica Acta*, *879*, 10–23. 10.1016/j.aca.2015.02.012 26002472 10.1016/j.aca.2015.02.012

[CR100] Oberbach, A., Blüher, M., Wirth, H., Till, H., Kovacs, P., Kullnick, Y., Schlichting, N., Tomm, J. M., Rolle-Kampczyk, U., Murugaiyan, J., Binder, H., Dietrich, A., & von Bergen, M. (2011). Combined proteomic and metabolomic profiling of serum reveals association of the complement system with obesity and identifies novel markers of body fat mass changes. *Journal of Proteome Research,**10*, 4769–4788. 10.1021/pr2005555 10.1021/pr2005555 21823675

[CR101] Park, S., Sadanala, K. C., & Kim, E-Y. (2015). A metabolomic approach to understanding the metabolic link between obesity and diabetes. *Molecules and Cells,**38*, 587–596. 10.14348/molcells.2015.0126 10.14348/molcells.2015.0126 PMC4507023 26072981

[CR12] Pottel, H., Vrydags, N., Mahieu, B., Vandewynckele, E., Croes, K., & Martens, F. (2008). Establishing age/sex related serum creatinine reference intervals from hospital laboratory data based on different statistical methods. *Clinica Chimica Acta*, *396*, 49–55. 10.1016/j.cca.2008.06.017 10.1016/j.cca.2008.06.017 18621041

[CR13] Pottel, H., Hoste, L., Delanaye, P., Cavalier, E., & Marten, F. (2012). Demystifying ethnic/sex differences in kidney function: Is the difference in (estimating) glomerular filtration rate or in serum creatinine concentration? *Clinica Chimica Acta*, *413*, 1612–1617. 10.1016/j.cca.2012.04.034 10.1016/j.cca.2012.04.034 22584028

[CR14] Seiffert, C., Khoshgoftaar, T. M., Van Hulse, J., & Napolitano, A. (2009). RUSBoost: A hybrid approach to alleviating class imbalance. *IEEE Transactions on Systems Man and Cybernetics-Part A: Systems and Humans*, *40*, 185–197. 10.1109/TSMCA.2009.2029559

[CR15] Shen, X., Wang, C., Zhou, X., Zhou, W., Hornburg, D., Wu, S., & Snyder, M. P. (2024). Nonlinear dynamics of multi-omics profiles during human aging. *Nature Aging*, *4*, 1619–1634. 10.1038/s43587-024-00692-2 39143318 10.1038/s43587-024-00692-2 PMC11564093

[CR16] Sumner, L. W., Amberg, A., Barrett, D., Beger, R., Beale, M. H., Daykin, C., Fan, T. W. M., Fiehn, O., Goodacre, R., Griffin, J. L., Hardy, N., Higashi, R., Kopka, J., Lindon, J. C., Lane, A. N., Marriott, P., Nicholls, A. W., Reily, M. D., & Viant, M. (2007). Proposed minimum reporting standards for chemical analysis. *Metabolomics*, *3*, 211–221. 10.1007/s11306-007-0082-2 24039616 10.1007/s11306-007-0082-2 PMC3772505

[CR17] Sun, G. W., Shook, T. L., & Kay, G. L. (1996). Inappropriate use of bivariable analysis to screen risk factors for use in multivariable analysis. *Journal of Clinical Epidemiology*, *49*, 907–916. 10.1016/0895-4356(96)00025-x 8699212 10.1016/0895-4356(96)00025-x

[CR18] Troyanskaya, O., Cantor, M., Sherlock, G., Brown, P., Hastie, T., Tibshirani, R., Botstein, D., & Altman, R. (2001). Missing value estimation methods for DNA microarrays. *Bioinformatics,**17*, 520–525. 10.1093/bioinformatics/17.6.520 11395428 10.1093/bioinformatics/17.6.520

[CR19] Xu, Y., & Goodacre, R. (2018). On splitting training and validation set: A comparative study of cross-validation, bootstrap and systematic sampling for estimating the generalization performance of supervised classification model. *Journal of Analysis and Testing*, *2*, 249–262. 10.1007/s41664-018-0068-2 30842888 10.1007/s41664-018-0068-2 PMC6373628

[CR20] Xu, Y., & Goodacre, R. (2025). Mind your Ps and Qs—Caveats in metabolomics data analysis. *Trends in Analytical Chemistry*, *183*, 118064. 10.1016/j.trac.2024.118064

[CR21] Zelena, E., Dunn, W. D., Broadhurst, D., Francis-McIntyre, S., Carroll, K. M., Begley, P., O’Hagan, S., Knowles, J. D., Halsall, A., Consortium, H. U. S. E. R. M. E. T., Wilson, I. D., & Kell, D. B. (2009). Development of a robust and repeatable UPLC – MS method for the long-term metabolomic study of human serum. *Analytical Chemistry,**81*(4), 1357–1364. 10.1021/ac8019366 19170513 10.1021/ac8019366

