# Supplementary material for: Combining clinical chemistry with metabolomics for metabolic phenotyping at population levels
Source: Metabolomics. 2025 Aug 29;21(5):126. doi: 10.1007/s11306-025-02331-2 (PMC12397149; doi:10.1007/s11306-025-02331-2)
Supplement: Supplementary file 1 — Supplementary Material 1 [file 11306_2025_2331_MOESM1_ESM.docx]

**SUPPORTING INFORMATION**

**Combining clinical chemistry with metabolomics for metabolic phenotyping at population levels**.

Yun Xu ^1^, Ian D Wilson^1,2^ Royston Goodacre^1^

^1^ Centre for Metabolomics Research, Department of Biochemistry, Cell and Systems Biology, Institute of Systems, Molecular and Integrative Biology, University of Liverpool, BioSciences Building, Crown St., Liverpool, L69 7ZB, United Kingdom

^2^Division of Systems Medicine, Department of Metabolism, Digestion and Reproduction, Imperial College London, Hammersmith Campus, London W12 0NN, United Kingdom

Table S1: The p-values and false discovery rates of the clinical chemistry variables between sex, age and BMI differences.

|  | Sex | | Age (< 50 vs. > 65) | | BMI (< 25 vs. >30) | |
| --- | --- | --- | --- | --- | --- | --- |
| Name | *p*-values | FDR | *p*-values | FDR | *p*-values | FDR |
| SBP | **7.74E-20^*^** | **7.35E-19** | **4.41E-20** | **8.38E-19** | **6.20E-15** | **3.00E-14** |
| DBP | **6.48E-15** | **3.88E-14** | **6.57E-06** | **2.65E-05** | **7.22E-25** | **6.85E-24** |
| PROT | **1.90E-13** | **7.21E-13** | **0.002372** | **0.005008** | 0.038163 | 0.051792 |
| CREA | **1.04E-89** | **1.97E-88** | 0.967148 | 0.967148 | 0.036884 | 0.051792 |
| SGLUC | 1.01E-11 | 2.73E-11 | 2.90E-05 | 9.17E-05 | 0.000367 | 0.000634 |
| SODIUM | 0.100407 | 0.112219 | 0.007798 | 0.014817 | 0.971466 | 0.971466 |
| K | 1.69E-05 | 2.67E-05 | 0.704303 | 0.743431 | 0.355087 | 0.396861 |
| CALCIUM | 0.279983 | 0.279983 | 0.072145 | 0.11423 | 0.433075 | 0.457135 |
| CHOL | 0.081292 | 0.096534 | **1.19E-06** | **7.53E-06** | 4.32E-05 | 8.21E-05 |
| TRIG | 5.07E-13 | 1.60E-12 | 0.243391 | 0.289027 | 3.10E-06 | 8.41E-06 |
| HDLC | 2.95E-09 | 5.60E-09 | **0.224288** | **0.284098** | **7.06E-28** | **1.34E-26** |
| LDLC | 0.229306 | 0.242046 | **6.98E-06** | **2.65E-05** | 1.29E-05 | 2.73E-05 |
| RATIO | **8.17E-15** | **3.88E-14** | **0.110564** | **0.150051** | **6.31E-15** | **3.00E-14** |
| UREA | 1.86E-05 | 2.71E-05 | 1.17E-07 | 1.11E-06 | 0.117915 | 0.140024 |
| TBIL | 3.45E-05 | 4.69E-05 | 0.017619 | 0.030433 | 0.09641 | 0.122119 |
| ALP | 2.64E-10 | 6.27E-10 | 7.38E-05 | 0.0002 | 4.97E-06 | 1.18E-05 |
| ALT | 5.07E-10 | 1.07E-09 | **0.000235** | **0.000557** | 9.03E-09 | 2.86E-08 |
| AST | 0.029483 | 0.037345 | 0.1055 | 0.150051 | 0.036344 | 0.051792 |
| GGT | 1.02E-06 | 1.76E-06 | 0.527361 | 0.589404 | 8.65E-12 | 3.29E-11 |

^*^: Bold font indicates this feature obtained V.I.P. scores > 1 in PLS-R/PLS-DA modelling

Clinical chemistry

Figure S1: The classification results of SVM models using clinical chemistry data

Clinical chemistry

Figure S2: The classification results of random forest models using clinical chemistry data

Figure S3: The classification results of SVM models using GC-MS data

GC – MS

GC – MS

Figure S4: The classification results of random forest models using GC-MS data

GC – MS

Figure S5: The classification results of PLS-DA models using GC-MS data

LC – MS Positive mode

Figure S6: The classification results of SVM models using LC-MS positive model data

Figure S7: The classification results of random forest models using LC-MS positive model data

LC – MS Positive mode

LC – MS Positive mode

Figure S8: The classification results of PLS-DA models using LC-MS positive model data

LC – MS Negative mode

Figure S9: The classification results of SVM models using LC-MS negative model data

LC – MS Negative mode

Figure S10: The classification results of random forest models using LC-MS positive model data

LC – MS Negative mode

Figure S11: The classification results of PLS-DA models using LC-MS negative model data

GC – MS + Clinic chemistry

Figure S12: The classification results of SVM models using GC-MS data fused with clinical chemistry data

GC – MS + Clinic chemistry

Figure S13: The classification results of random forest models using GC-MS data fused with clinical chemistry data

GC – MS + Clinic chemistry

Figure S14: The classification results of PLS-DA models using GC-MS data fused with clinical chemistry data

LC – MS Positive mode + Clinic chemistry

Figure S15: The classification results of SVM models using LC-MS positive mode data fused with clinical chemistry data

LC – MS Positive mode + Clinic chemistry

Figure S16: The classification results of random forest models using LC-MS positive mode data fused with clinical chemistry data

LC – MS Positive mode + Clinic chemistry

Figure S17: The classification results of PLS-DA models using LC-MS positive mode data fused with clinical chemistry data

LC – MS Negative mode + Clinic chemistry

Figure S18: The classification results of SVM models using LC-MS negative mode data fused with clinical chemistry data

LC – MS Negative mode + Clinic chemistry

Figure S19: The classification results of random forest models using LC-MS negative mode data fused with clinical chemistry data

LC – MS Negative mode + Clinic chemistry

Figure S20: The classification results of PLS-DA models using LC-MS negative mode data fused with clinical chemistry data

Figure S21: Learning curves of random forest models

Figure S22: Learning curv es of SVM models

Figure S23: PLS-R for age prediction using single data set

Median *Q*^2^ = 0.3423, *p* < 0.001

Clinical chemistry

GC-MS

Median *Q*^2^p= 0.1460, *p* < 0.05 (0.0346)

Median *Q*^2^ = 0.279, *p* < 0.001

LC-MS, Positive mode

Median *Q*^2^ = 0.2638, p < 0.001

LC-MS, Negative mode

LC-MS Pos + Clinical chemistry

Median Q2 = 0.4976, *p* < 0.001

LC-MS Neg + Clinical chemistry

Median Q2 = 0.5105, p < 0.001

GC-MS + Clinical chemistry

Median Q2 = 0.4147, p < 0.001

Figure S24: PLSR for age prediction using metabolomics data fused with clinical chemistry data

Figure S25: PLS-R for BMI prediction using single data set

Median *Q*^2^ = 0.2943, *p* < 0.001

Clinical chemistry

LC-MS Positive mode

Median *Q*^2^ = 0.2057, *p* < 0.001

Median *Q*^2^ = 0.2029, *p* < 0.001

LC-MS negative mode

GC-MS

Median *Q*^2^ = 0.1388, *p* < 0.05 (0.0126)

Median *Q*^2^ = 0.3617, *p* < 0.001

LC-MS Neg. + Clinical chemistry

Median *Q*^2^ = 0.2656, *p* < 0.01 (0.001)

GC-MS + Clinical chemistry

LC-MS Pos. + Clinical chemistry

Median *Q*^2^ = 0.3167, p < 0.001

Figure S26: PLSR for age prediction using metabolomics data fused with clinical chemistry data
